# Understanding the Functional Plasticity in Neural Networks of the Basal Ganglia in Cocaine Use Disorder: A Role for Allosteric Receptor-Receptor Interactions in A2A-D2 Heteroreceptor Complexes

**DOI:** 10.1155/2016/4827268

**Published:** 2016-10-30

**Authors:** Dasiel O. Borroto-Escuela, Karolina Wydra, Julia Pintsuk, Manuel Narvaez, Fidel Corrales, Magdalena Zaniewska, Luigi F. Agnati, Rafael Franco, Sergio Tanganelli, Luca Ferraro, Malgorzata Filip, Kjell Fuxe

**Affiliations:** ^1^Department of Neuroscience, Karolinska Institutet, Retzius väg 8. 17177 Stockholm, Sweden; ^2^Department of Biomolecular Science, Section of Physiology, University of Urbino, Campus Scientifico Enrico Mattei, Via Ca' le Suore 2, I-61029 Urbino, Italy; ^3^Observatorio Cubanos de Neurociencias, Grupo Bohío-Estudio, Yaguajay, Cuba; ^4^Laboratory of Drug Addiction Pharmacology, Institute of Pharmacology, Polish Academy of Sciences, 12 Smetna Street, PL-31-343 Kraków, Poland; ^5^Institute of Biomedicine and Translational Medicine, Department of Physiology, University of Tartu, 19 Ravila Street, 50411 Tartu, Estonia; ^6^Universidad de Málaga, Instituto de Investigación Biomédica de Málaga, Facultad de Medicina, Málaga, Spain; ^7^Centro Nacional de Neurociencias, La Habana, Cuba; ^8^Departament de Bioquímica i Biomedicina Molecular, Facultat de Biologia, Universitat de Barcelona, Barcelona, Spain; ^9^Centro de Investigaciones en Red, Enfermedades Neurodegenerativas (CIBERNED), Madrid, Spain; ^10^Department of Life Sciences and Biotechnology, University of Ferrara, Ferrara, Italy; ^11^Department of Medical Sciences, University of Ferrara, Ferrara, Italy

## Abstract

Our hypothesis is that allosteric receptor-receptor interactions in homo- and heteroreceptor complexes may form the molecular basis of learning and memory. This principle is illustrated by showing how cocaine abuse can alter the adenosine A2AR-dopamine D2R heterocomplexes and their receptor-receptor interactions and hereby induce neural plasticity in the basal ganglia. Studies with A2AR ligands using cocaine self-administration procedures indicate that antagonistic allosteric A2AR-D2R heterocomplexes of the ventral striatopallidal GABA antireward pathway play a significant role in reducing cocaine induced reward, motivation, and cocaine seeking. Anticocaine actions of A2AR agonists can also be produced at A2AR homocomplexes in these antireward neurons, actions in which are independent of D2R signaling. At the A2AR-D2R heterocomplex, they are dependent on the strength of the antagonistic allosteric A2AR-D2R interaction and the number of A2AR-D2R and A2AR-D2R-sigma1R heterocomplexes present in the ventral striatopallidal GABA neurons. It involves a differential cocaine-induced increase in sigma1Rs in the ventral versus the dorsal striatum. In contrast, the allosteric brake on the D2R protomer signaling in the A2AR-D2R heterocomplex of the dorsal striatopallidal GABA neurons is lost upon cocaine self-administration. This is potentially due to differences in composition and allosteric plasticity of these complexes versus those in the ventral striatopallidal neurons.

## 1. Introduction

The receptor-receptor interaction field began with the studies on neuropeptide-monoamine receptor-receptor interactions in brain membrane preparations which altered especially the affinity of the monoamine receptor subtypes [1, 2]. The aim of the study was to understand the integration of peptide and monoamine signals present in many neuronal networks of the Central Nervous System (CNS). It was proposed that they took place in postulated heteroreceptor complexes in the plasma membrane [3]. In these complexes, adaptor proteins can participate and higher-order homo- and heteroreceptor complexes can also exist in which allosteric receptor-receptor interactions operate [4–8]. We call them hetero- or homoreceptor complexes since a number of other proteins also participate like adaptor proteins which can directly bind to the receptor protomers undergoing homomerization or heteromerization [8–13].

These integrative mechanisms give sophisticated dynamics to the structure and function of the heteroreceptor complexes with regard to alterations of recognition, signaling, and trafficking of the receptor protomers. Due to allosteric receptor-receptor interactions, increased diversity and selectivity develop in receptor pharmacology and function [19, 20]. Dynamics is also given to the heteroreceptor complex through its balance with the corresponding homoreceptor complexes and other types of heteroreceptor complexes which share one or more receptor protomers [8, 10, 11].

It is of high interest to understand the role of the allosteric receptor-receptor interactions in heteroreceptor complexes in neurons, brain circuits, and at the global network level. In the current paper, we will discuss how this novel biological principle can serve to modulate the plasticity and function of the neural-glial networks of the CNS. The major focus will be on how cocaine abuse in rats can alter the adenosine A2A receptor- (A2AR-) dopamine D2 receptor (D2R) heterocomplexes and their receptor-receptor interactions in the neural-glial networks of the basal ganglia [12, 21].

## 2. Integration of Volume Transmission and Synaptic Transmission in the Neural-Glial Networks of the CNS through Receptor-Receptor Interactions in Heteroreceptor Complexes

The G protein-coupled receptor (GPCR) heterocomplex concept includes not only GPCR-GPCR heterocomplexes but also the interactions between GPCR and other membrane receptors, for instance, the ion channel receptors or receptor tyrosine kinases (RTK) heterocomplexes [9, 22–29]. GPCRs with high affinity for neurotransmitters are major receptors for volume transmission (VT) and ion channel receptors like NMDA, AMPA, and GABA receptors are major receptors for synaptic transmission. Therefore, GPCR-ion channel receptor heterocomplexes are major integrators of VT and synaptic transmission in neural-glial networks [30, 31]. This integration, which takes place at both the prejunctional and postjunctional level, can up- or downregulate synaptic efficacy and thus synaptic strength within neural-glial networks. The VT signals may originate, for example, from local glial cells, extrasynaptic release of transmitters from neural afferents, local collaterals, and soma-dendrites and from diffusion and flow of for example, neuropeptides, and proteins or of extracellular vesicles from cerebrospinal fluids and/or surrounding neural-glial networks also forming functional modules. This major mechanism for the balance and integration of synaptic and volume transmission may then lead to changes in the preferred outputs from the neural-glial module which in turn will change the function in the brain circuit involved.

Instead, GPCR-RTK heterocomplexes are of relevance for the survival and repair of the neural-glial networks by integrating and balancing trophic signaling with the GPCR-mediated metabolic signaling and information handling [32]. Relevant examples of direct GPCR-RTK receptor-receptor interactions are A2AR-FGFR1 [25, 33], 5-HT1A-FGFR1 [9, 27, 28], M3R-FGFR1 [34], TGR5-EGFR [35], and ADRB2-INR heteroreceptor complexes [36]. Wiese et al. [37] and Assaife-Lopes et al. [38] demonstrated transactivation mechanisms between adenosine A2A receptor and TrkB receptor, which can involve allosteric mechanisms via direct GPCR-RTK interactions.

The changes in the protomer signaling panorama within the heteroreceptor complex may be highly dynamic processes related to the transient nature of the receptor complexes formed and can represent learning if by repetition it leads to short-term memory (see below) [13, 31].

## 3. Understanding the Molecular Basis of Learning and Memory through Allosteric Receptor-Receptor Interactions in Multiple Homo- and Heteroreceptor Complexes in Neuronal Networks

**Figure 1:**
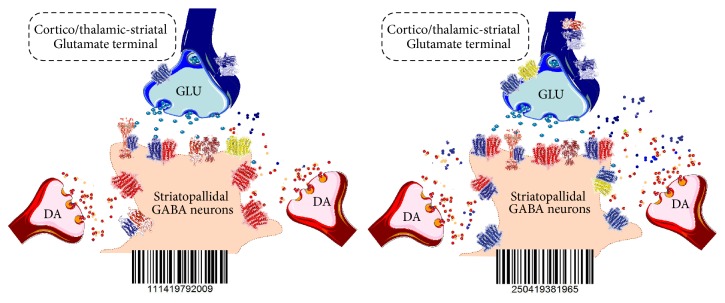
Illustration of possible reorganization of the homo- and heteroreceptor complexes within the synaptic and extrasynaptic regions of the glutamate synapses of the striatopallidal GABA neurons in the learning process. The receptor monomers are not illustrated. The left panel represents the basal state and the right panel the state that has developed in the learning process. They can become stabilized into a long lasting receptor assemblies through the formation of novel adaptor and scaffolding proteins. If so, they will represent short-term memory that can be converted into long-term memory through further consolidation.

Changes in synaptic efficacies via alterations in synaptic strength are regarded as fundamental to learning [39]. The theory was recently advanced that such changes in synaptic plasticity can be brought about by anatomical reorganization of multiple postsynaptic homo- and heteroreceptor complexes and by resetting the multiple allosteric receptor-receptor interactions according to the patterns and amounts of synaptic signals released from the synaptic terminals decoded from the arriving patterns of action potentials [13, 31]. Such a molecular reorganization of the receptor-protein architecture of the entire postsynaptic membrane is proposed to be the molecular basis of learning and short-term memory. It may be described as a change of the barcode in the individual synapse, which leads to the formation of a new molecular engram. According to the theory, a reorganization of presynaptic homo- and heteroreceptor complexes also developed in the learning and memory process in order to ensure the maintenance of the pattern and amounts of multiple transmitters and modulators released which must be sensed by the postsynaptic membrane (Figure 1).

Previous work discussed how GPCR modulates the kinetics of G protein signaling [40]. The signaling dynamics of 5-HT1A-FGFR1 and D2-NTS1 heteroreceptor complexes were also reported [26, 41]. Oligomer size of diffusing GPCRs in the plasma membrane can be determined by fluorescence correlation spectroscopy with photon counting histogram analysis and the 5-HT2C receptor diffused as a dimer [42]. Single fluorescent-molecule imaging demonstrates dynamic GPCR dimerization using a chemoattractant GPCR, the N-fomyl peptide receptor (FPR) [43]. A number of cycles of monomer and homodimer formation take place over one second. Thus, FPR dimers are quickly formed from FPR monomers and FPR monomers are rapidly formed from rapid dissociation of the FPR dimers [44]. Experimental studies support the view that the dynamic properties of GPCRs depend on their coupling to the membrane environment, where the lipid composition plays a role in ligand-induced properties [45]. The dynamics are also indicated from studies using spatial intensity distribution analysis [46] and RT-BRET to understand the oligomeric organization of GPCRs [26]. The analysis demonstrated multiple forms from monomers to higher-order oligomers of the GPCR studied.

These studies support our view that the dynamic panorama of homo- and heteroreceptor complexes and their stabilization can form the basis of learning and memory at the molecular level and represent the molecular engram, when consolidated (Figure 1).

Our hypothesis on the molecular basis of learning and memory is summarized as follows:

- Learning requires a new temporal qualitative and quantitative pattern of release of neurotransmitters. The transient reorganization of the homo- and heteroreceptor complexes in the postsynaptic membrane contributes to pattern formation.
- This molecular reorganization of the postsynaptic membrane leads to a novel barcode, which represents a short-term memory (transient molecular engram) and defines the new pattern of transmitter release.
- This memory process is facilitated by the reorganization also of the presynaptic receptor complexes to help maintain the new transmitter release pattern to be learned by the postsynaptic membrane. Thus, the altered transmitter panorama can change the formation or disrupt the presynaptic receptor complexes through agonist dependent processes. A retrograde feedback from the new postsynaptic transient molecular engram can also assist in this process. It can involve the release and diffusion of soluble factors like purines and trophic factors and of extracellular vesicles like exosomes and microvesicles containing proteins including receptors and lipids.
- The consolidated reorganization of the postsynaptic receptor complexes and their extrasynaptic regions, including changes in scaffold proteins like Disc1 and SHANK, can lead to long-term memory. The long-term memory should result in a barcode and molecular engram similar to one found in short-term memory. The mechanism for this process may involve the transformation of parts of the heteroreceptor complexes into unique transcription factors which can lead to the formation of novel adaptor proteins like GPCR interacting proteins and receptor activity-modifying proteins. Some of these proteins can link the heteroreceptor complexes more strongly together. Others can link the heteroreceptor complexes more strongly to the cytoskeleton and to scaffold proteins and synaptic cell adhesion molecules.
- Of special interest for the formation of truly long-term molecular engrams may be the release of transmitters from close-by terminals belonging to emotional brain circuits using, for example, dopamine (DA) as a transmitter [31]. When such terminal networks are activated due to motivation and reward linked to the learning and memory process, the transmitter, for example, DA, can interact with the synapse, where the molecular engram is being formed, through short distance volume transmission. It is postulated that the activation of, for example, synaptic and extrasynaptic DA heteroreceptor complexes on the postsynaptic side has an ability via unique transcription factors to produce special adaptor proteins that can bind to the receptor-protein architecture of the molecular engram and consolidate them with an exceptional efficacy leading to life-long memories.

## 4. On the Role of Antagonistic A2A-D2 Allosteric Receptor-Receptor Interactions in Heteroreceptor Complexes for Neuronal Plasticity: Studies in Cocaine Use Disorder Models

It was early on observed that the adenosine A2AR agonist CGS 21680 reduced the affinity of the dopamine D2R agonist binding sites, especially in the high affinity state, in dorsal striatum and nucleus accumbens [47, 48]. Thus, antagonistic A2AR-D2R interactions appear to exist in the plasma membrane of neurons. It was further demonstrated that the A2ARs and D2Rs coaggregate, cointernalize, and codesensitize in heteroreceptor complexes [49]. In line with these findings, agonist activation of the A2AR protomer in the A2AR-D2R heteroreceptor complexes reduces D2R Gi/o-mediated signaling through the allosteric receptor-receptor interaction [19, 50]. Instead, the A2AR agonist increases the D2R beta-arrestin2 mediated signaling and can be characterized as a biased inhibitory modulator of the Gi/o-mediated D2R signaling [51]. This action may explain its atypical antipsychotic activities [50, 52].

The A2AR-D2R heterocomplexes interface involves mainly intracellular domains where electrostatic interactions form clasps in the interface (hot spots) [53–55] and transmembrane domains [56] where triplet amino acid homologies can play a special role as adhesive guides in the formation of the heteromer [57].

The overall architecture of the global GPCR heterodimer network (http://www.gpcr-hetnet.com/) [58] demonstrates that both A2ARs and D2Rs are hub components, each able to interact with ten or more different GPCRs. Thus, the A2AR-D2R heteroreceptor complex in a neuron can be in a complex balance not only with A2AR and D2R homoreceptor complexes but also with other types of A2AR and D2R heteroreceptor complexes, respectively [8, 10, 11].

The A2AR-DR2 heteroreceptor complexes were demonstrated in the dorsal and ventral striatum by means of the proximity ligation assay (*in situ* PLA) [34, 59]. In relation to cocaine use disorder, it is of substantial interest that these complexes may participate in postjunctional mesolimbic DA transmission in the nucleus accumbens core and shell in view of their presence in high densities in these two regions.

*In situ* PLA-positive A2AR-D2R heteroreceptor complexes are visualized in striatal sections as red clusters (blobs, puncta) within different regions of the neuropil of caudate putamen and nucleus accumbens core and shell, which can be quantitated as, for example, average number of blobs/cell [34].

### 4.1. Ventral Striatopallidal GABA Neurons

A2AR-D2R heteroreceptor complexes exist mainly in the ventral striatopallidal GABA neurons that mediate antireward [60]. The circuit continues from the ventral pallidum into the medial thalamic nucleus-prefrontal cortex as well as into the ventral midbrain, there mainly appearing as collaterals from the other regions [61]. The stoichiometry of these complexes is unknown but can involve heterotrimers, heterotetramers, and etcetera. Also the critical balance with other types of A2AR or D2R heteroreceptor complexes is unknown [11]. One ventral striatopallidal GABA neuron may differ from another one with regard to the stoichiometry and composition of the A2AR-D2R heteroreceptor complexes as well as their distribution pattern among synapses. Does each neuron of this pathway possess a unique distribution of A2AR-D2R heteroreceptor complexes to certain of its synapses while others lack these complexes? Can the switching in the balance between different D2R and A2AR heteroreceptor complexes in the same synapse be part of the learning process and contribute to short term and, if relevant, long-term memory and thus to the formation of a molecular engram (see above)? It is also of special interest that a loss of TrkB, the receptor for BDNF, in D2R positive accumbens neurons leads to increased neuronal excitability and suppression of reward [60]. These results open up the possibility that TrkB-D2R heteroreceptor complexes exist in which TrkB directly or indirectly make the inhibitory Gi/o-mediated D2R signaling possible, which leads to hyperpolarization and reduced excitability and firing. Thus, it appears possible that A2AR-D2R-TrkB heteroreceptor complexes can also exist in this antireward system. In fact, it was found that A2ARs are necessary for the baseline BDNF levels and for enhancement of synaptic transmission by BDNF as determined in the hippocampus [62].

### 4.2. Pharmacological Studies with Cocaine Self-Administration Procedures

We have studied the effects of the A2AR agonist CGS 21680 on cocaine self-administration under different schedules of reinforcement. Active lever presses and cocaine infusions were attenuated by CGS 21680 [63] and these effects could not be explained by sedation and reduction in locomotion although such inhibitory motor actions are linked to A2AR activation [63]. These results clearly indicated that activation of A2ARs inhibits cocaine-induced reward in agreement with previous work [21, 64]. Of substantial interest were the inhibitory effects of the CGS 21680 treatment in a progressive ratio schedule of reinforcement protocol with a 4-hour breaking point [63] indicating that A2AR stimulation brings down not only cocaine reward but also cocaine motivation.

A neurochemical correlate was also obtained. The effects of an accumbal perfusion of CGS 21680 were studied on changes induced by a 2 h cocaine self-administration session on extracellular accumbal DA, GABA, and glutamate levels. In the cocaine self-administration session over 2 h, CGS 21680 induced an increase in extracellular GABA levels which appeared to reduce the increase in extracellular DA levels found during cocaine self-administration [63]. This mechanism may restore the glutamate drive to the prefrontal cortex from the dorsomedial thalamic nucleus [50] helping the prefrontal cortex to restore its control of the accumbens reward system. These results are compatible with the involvement of antagonistic allosteric A2AR-D2R interactions in the ventral striatopallidal antireward system in mediating the A2AR induced inhibition of cocaine reward [63]. Thus, restoring the allosteric antagonistic plasticity in the neuronal A2AR-D2R heteroreceptor complex may contribute to anticocaine actions of A2AR agonists.

Such an increase in antagonistic allosteric plasticity in the A2AR-D2R heteroreceptor complex can also help explain why the A2AR agonist CGS 21680 produced a dose-dependent blockade of the cocaine, quinpirole, and cue-induced reinstatement of cocaine seeking [65, 66]. In support of the view that the antagonistic A2AR-D2R interactions may control drug seeking, it was found that CGS 21680 was 4 times more powerful in counteracting the D2-like receptor agonist quinpirole-induced relapse versus cocaine-induced relapse.

These inhibitory actions of CGS 21680 towards cocaine- or quinpirole-induced reinstatement of cocaine seeking behaviors in rats were in line with previous studies using i.p. injections or intra-accumbal infusions with the A2AR agonist [67]. CGS 21680 also blocked cue-induced food seeking [65]. It is of relevance to point out that there exist also targets in the nucleus accumbens for A2AR agonists other than the A2AR-D2R heteroreceptor complexes like the A1-A2A heteroreceptor complexes preferentially located on the corticostriatal glutamate terminals [68, 69]. Here, A2AR agonists can enhance glutamate release inter alia by an antagonistic allosteric receptor-receptor interaction with the A1R protomer which reduces its brake on glutamate release, which can modulate the activity of the D2R positive antireward and D1R positive reward neurons. Furthermore, there is evidence for the existence of postjunctional A2AR-mGlu5R and A2AR-D2R-mGlu5R heteroreceptor complexes in the striatopallidal GABA neurons [69, 70] where the mGlu5R protomer synergizes with the A2AR protomer to reduce D2R protomer signaling and its inhibition of the striatopallidal GABA neurons [71]. By targeting also these heterocomplexes, the anticocaine actions of A2AR agonists can become amplified.

It is also possible that these two types of A2AR-mGlu5R heteroreceptor complexes exist in the glutamate nerve terminals where the A2ARs and mGlu5Rs colocalize and functionally interact in synergistically enhancing glutamate release [72]. It appears that A2AR agonists by targeting several A2AR heteroreceptor complexes and A2AR homoreceptor complexes in the nucleus accumbens can increase activity in the antireward GABA neurons leading to anticocaine actions. It was proposed that A2AR participates in a mechanism to integrate the GABA, DA, and glutamate signaling [73]. In this mechanism, also A2AR homoreceptor complexes participate and can inter alia be involved at the presynaptic level in increasing the synaptic striatopallidal GABA transmission in the globus pallidus [74].

Underlining the existence of an endogenous adenosine tone reducing D2R signaling, A2AR antagonists KW 6002 and SCH 58261 induce reinstatement of cocaine seeking behavior in rats extinguished from cocaine self-administration. The mechanism is proposed to involve a blockade of the A2AR protomer of the neuronal A2AR-D2R heteroreceptor complex in the ventral striatopallidal GABA pathway. By altering the allosteric interactions in this A2AR-D2R heteroreceptor complex, the brake on D2R signaling is reduced and cocaine seeking can develop due to reduction of the glutamate drive to the prefrontal cortex [50]. In this way, it is possible that cognitive control including flexible goal-directed behavior probably becomes diminished and cocaine seeking develops. D2Rs are known to have a key role in cocaine use disorder [75, 76]. However, A2ARs appear to have a different role in the dorsal striatum where a striatum-specific A2AR deletion led to selective impairment of habit formation [77]. Later on, it was demonstrated that dorsomedial striatopallidal GABA neurons upon optogenetic activation of their A2ARs diminished goal-directed behavior [78]. Thus, in this circuit, A2AR signaling when linked to reward may favour habit formation.

It is clear, however, that A2AR agonists target both A2AR homo- and A2AR-D2R heteroreceptor complexes and that A2AR homoreceptor complexes can contribute to the anticocaine actions observed. There are at the moment no preferential A2AR agonists for the A2AR protomer of the A2AR-D2R heteroreceptor complex nor preferential antagonists for the D2R protomer of this complex. Multitargeting drugs with A2AR agonist [79] and D2R antagonist activities are promising molecules to combat cocaine use disorder.

Heterobivalent drugs are attractive novel drugs to be developed [80–83]. In this case, they should be built based on an A2AR agonist pharmacophore linked to a D2R antagonist pharmacophore which should preferentially target A2AR-D2R heteroreceptor complexes with a high affinity and selectivity. Their selective action on neurons containing A2AR-D2R heteroreceptor complexes may predict less side effects. Previously, A2AR antagonist-D2R agonist bivalent compounds were proposed as tools to find A2AR-D2R receptor heteromers and as a novel strategy for treatment of Parkinson's disease [84].

Small inhibitory interface peptides targeting the A2AR-D2R interface are also of interest as they may block the formation of the A2AR-D2R heteroreceptor complex or alter the heteroreceptor-mediated signaling [56]. Inhibitory interface peptides were first used in studies on D5R-GABA-A [23], D1R-NMDAR [22], and D2R-NR2B [24] heterocomplexes showing their physiological role. Competitive peptides were also used in determining the physiological role of A2AR-D2R heterodimerization in regulating NMDAR mediated excitation of neurons in the nucleus accumbens [85]. Furthermore, their use could help to unravel the role of the A2AR-D2R heteroreceptor complexes versus the A2AR homoreceptor complex in the actions of A2AR agonists on cocaine reward and cocaine seeking.

### 4.3. Allosteric A2AR-D2R Interactions in Cocaine Self-Administration

Previous work demonstrated that antagonistic allosteric A2AR-D2R interactions exist in the ventral and dorsal striatum of drug naïve control rats involving an A2AR induced reduction of the affinity of the high affinity D2R agonist binding site [47, 48]. It was therefore of high interest to study how maintenance of cocaine-self-administration affected this allosteric interaction in the ventral and dorsal striatum [16]. It was found that cocaine self-administration differentially affected the allosteric A2AR-D2R interactions in the ventral and dorsal striatum versus the yoked saline group.

The current protocol produces elevation of extracellular levels of DA in the nucleus accumbens in 2 h cocaine self-administration sessions [86] and no changes in the A2AR and D2R antagonist binding sites in the striatum were observed in terms of *B*_max_ and *K*_*d*_ values [87].

It was found that CGS 21680, the A2AR agonist,* ex vivo* using 3H-raclopride/quinpirole competition curves produced a significant reduction of the affinity of the D2-like high affinity receptor agonist binding site after cocaine self-administration in the ventral striatum versus the yoked saline group. The mechanism may involve a cocaine-induced change in the balance of A2AR-D2R heteroreceptor complexes versus A2AR homoreceptor complexes and other types of A2AR heteroreceptor complexes favouring an increase in A2AR-D2R heteroreceptor complexes. Such a change can contribute to the increase in the antagonistic allosteric A2AR-D2R interaction observed.

Our hypothesis is that changes in neuronal plasticity in cocaine self-administration may involve a reorganization of the balance of the A2AR monomers and homo/heteroreceptor complexes which can vary from one synapse to another and from one neuron to the other one. Several A2AR heteroreceptor complexes were already discussed and can be present at the pre- and/or postsynaptic level including the A2AR-D2R heteroreceptor complexes and together with other heteroreceptor complexes like the NMDAR heteroreceptor complexes [22, 24]. It should be noted that inactivation of A2ARs blocks long-term potentiation [88] and working memory deficits are reversed upon inactivation of A2ARs [89]. A2ARs can also form heterocomplexes with FGFR1 [25, 33].

Another mechanism may be that cocaine through its release of DA and/or indirect/direct allosteric enhancement of D2R signaling [90] can induce an allosteric feedback to help reduce D2R function via this antagonistic allosteric mechanism in the ventral striatum [16]. Thus, the anticocaine actions of A2AR agonists may at least in part be determined by the strength of the antagonistic allosteric A2AR-D2R interaction and the number of A2AR-D2R heteroreceptor complexes present, which leads to increased activity of the ventral striatopallidal GABA neurons representing an antireward system.

Dynamic changes also developed in the allosteric A2AR-D2R interactions in the dorsal striatum upon maintenance of cocaine self-administration. However, in this case, they resulted in disappearance of the antagonistic A2AR-D2R interaction found in yoked saline rats after* ex vivo* incubation of the membrane preparations with the A2AR agonist CGS 21680 [16]. As a consequence, no reduction of the affinity in the D2R high affinity agonist binding site was observed. Thus, the allosteric brake on the D2R protomer signaling in the A2AR-D2R heteroreceptor complex of the dorsal striatopallidal GABA neurons is lost upon cocaine self-administration. This is potentially due to differences in composition and allosteric plasticity of these complexes versus those in the ventral striatopallidal neurons. In line with these results, it was previously found that DA increased its potency to increase D2R Gi/o coupling in dorsal but not ventral striatal membranes in cocaine self-administration [87]. It is proposed that the disappearance of the allosteric brake on the D2R protomer signaling in the A2AR-D2R heteroreceptor complex can alter cocaine seeking, habit forming learning, and/or locomotor sensitization which remains to be determined.

In view of the allosteric theory of learning and memory based on the formation of a molecular engram built up of homo- and heteroreceptor complexes [12, 13], it is proposed that the loss of the allosteric A2AR-D2R interactions is caused by a reorganization or loss of the A2AR-D2R heteroreceptor complex. This leads to an alteration of the molecular engram in the synapses affected linked to a change in learning due to loss of the allosteric brake. The altered long-term memories formed are likely the basis for the novel habits formed versus goal-directed behavior. Neuronal plasticity induced by cocaine at the molecular level through allosteric mechanisms can therefore produce changes in the brain circuits involved. According to the work of Li et al. [78], increases in A2AR signaling should lead to suppression of goal-directed behavior. Therefore, the increased balance of A2AR and D2R signaling in the dorsomedial striatopallidal GABA neurons through loss of the inhibitory allosteric mechanism should reduce, if anything, the appearance of abnormal habit formation.

The hypothesis is advanced that the mechanism for the potential therapeutic actions of A2AR agonists in cocaine addiction is their ability to activate the A2AR brake on D2R protomer signaling in the ventral striatum even more strongly in this disease than in drug naïve controls. This action will markedly reduce the rewarding actions of cocaine through the activation of the antireward system. However, the A2AR protomer brake on the D2R protomer signaling in the dorsal striatum in contrast disappears upon prolonged cocaine use. Its impact on cocaine seeking behavior remains to be determined.

The mechanism for the differential effects observed on the allosteric A2AR-D2R interactions in the ventral versus the dorsal striatum in cocaine self-administration can depend on regional differences in the stoichiometry and composition of the A2AR-D2R heteroreceptor complexes [10, 11]. This could involve differential recruitment of adaptor proteins and other receptors to the heteroreceptor complex.

## 5. On the Existence of Sigma-1R-D2R and A2AR-D2R-Sigma-1R Heteroreceptor Complexes in the Ventral and Dorsal Striatum and Their Potential to Understand the Differential Allosteric A2AR-D2R Receptor-Receptor Interactions in These Regions

**Figure 2:**
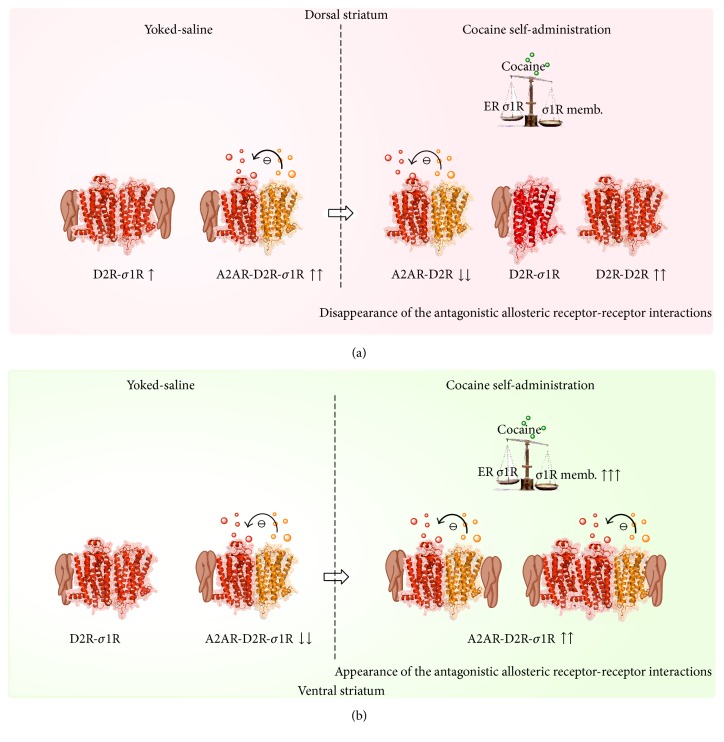
((a) Dorsal striatum)* Yoked saline group*. The high density of D2R-sigma1R [14, 15] leads to an increased formation of A2AR-D2R-sigma1R heterocomplexes which together with the A2AR-D2R heterocomplexes can be in dominance over D2 homoreceptor complexes. This can help explain the significant antagonistic A2AR-D2R interaction observed [16].* Cocaine self-administration (maintenance) group*. Cocaine can bind to the sigma1R [17] but does not increase its recruitment to the plasma membrane in the dorsal striatum [18]. Instead, it is proposed that the major change induced by cocaine can be the increase in extracellular DA levels which is postulated to increase the formation of D2R homoreceptor complexes. Due to competition, this can reduce the number of A2AR-D2R and A2AR-D2R-sigma1R heteroreceptor complexes. As a result, the antagonistic allosteric A2AR-D2R interactions disappear [16]. ((b) Ventral striatum)* Yoked saline group.* The lower density of D2R-sigma-1R, especially in nuc accumbens core [14, 15], may lead to the preferential formation of D2R-sigma1R heterocomplexes in view of a possible higher affinity of sigma1R for D2R monomer-homoreceptor complexes versus the affinity for the A2AR-D2R heteroreceptor complexes. Due to competition, the A2AR-D2R heteroreceptor complexes will become reduced and only weak nonsignificant antagonistic A2AR-D2R interactions develop.* Cocaine self-administration (maintenance) group*. Upon cocaine self-administration, cocaine can here increase sigma1R expression [18] and increase its recruitment to the plasma membrane where it interacts with D2R potentially at several interfaces. In view of the marked increase of sigma1R in the plasma membrane increased formation of A2AR-D2R-sigma1R heteroreceptor complexes can develop with restoration of significant antagonistic A2AR-D2R interactions [16] in spite of increases in extracellular DA levels postulated to favour D2R homodimerization.

It is known that sigma-1R-D2R heteroreceptor complexes exist and form higher-order oligomers and are specific for D2Rs versus D3Rs and D4Rs [17]. Recently, it was shown with BRET analysis that sigma-1Rs form heteroreceptor complexes to the same degree with D2 short and long receptor isoforms [14, 15]. It is of substantial interest that the sigma-1R addition in increasing concentrations does not reduce the BRET signal obtained with either D2 short or D2 long isoforms in cell models. The explanation can be multiple interfaces for sigma-1R-D2R interactions; that is, the sigma-1R may bind to different regions of the D2R [15]. With* in situ* PLA, the sigma-1R-D2R heteroreceptor complexes were demonstrated within discrete regions of the dorsal and ventral striatum of the rat [14, 15]. The densities of complexes were substantially higher in the dorsal versus the ventral striatum [14, 15] and a selective cocaine-induced increase of the sigma1Rs was demonstrated in the ventral versus the dorsal striatum [18]. These results can help understand the differential effects on allosteric A2AR-D2R interactions observed upon cocaine self-administration in the ventral and dorsal striatum (see above). The mechanism may involve an increased presence of A2AR-D2R-sigma-1R heteroreceptor complexes in the ventral versus the dorsal striatum after cocaine self-administration (Figure 2).

In relation to this hypothesis, there are novel data on the actions of cocaine at a low concentration on D2R signaling using CREB luciferase reporter gene assay in HEK293 cells. Cocaine (100 nM) increased the potency of D2R signaling when D2Rs and sigma-1Rs were expressed together [14]. Cocaine lacked effects when the D2Rs were expressed alone. Thus, cocaine at a low concentration can enhance the D2R mediated neurochemical effects like enhancement of Gi/o-mediated coupling but only in the sigma-1R-D2R heteroreceptor complex. These effects remained in the presence of DA transporter inhibitors and likely represented allosteric effects on the D2R protomers mediated via the sigma-1R in the heteroreceptor complex. In the absence of cocaine, a coexpression of D2Rs and sigma-1Rs show a reduced potency of D2R agonists to inhibit the CREB signal [14, 15]. It is of high interest that, even in the presence of 100 nM cocaine, CGS 21680 strongly reduced the potency of the D2R agonist to inhibit the CREB signal. This suggests the existence of a putative A2AR-D2R-sigma-1R heteroreceptor complex in HEK293 cells where the antagonistic A2AR-D2R interactions may be strongly enhanced. In this context, it is relevant to notice the partial overlap in the distribution of D2R-sigma1R and A2AR-D2R heteroreceptor complexes in the nucleus accumbens shell as found with* in situ* PLA [15].

In line with these results, it was found in HEK293 cells coexpressing sigma-1Rs and D2Rs that cocaine at 1 nM increased the *B*_max_ values of plasma membrane D2Rs, an action blocked by the sigma-1R antagonist PD 144418. The mechanism for the increase in the *B*_max_ values can involve a blockade of cocaine-induced D2R-YFP internalization when the sigma-1R forms a heteroreceptor complex with the D2R. This action was so far observed in the 1–100 nM range of cocaine [14, 15]. These results on cocaine actions in the nanomolar range on D2R-sigma-1R and on putative A2AR-D2R-sigma-1R heteroreceptor complexes are of high interest. They offer a new possibility to understand the differential effects of self-administered cocaine on the allosteric A2AR-D2R interactions in the dorsal versus the ventral striatum in the cocaine self-administration experiments (Figure 2).

It is proposed that the higher density of D2R/sigma-1R heterocomplexes in the dorsal versus the ventral striatum [14, 15] and selective cocaine-induced increases of the sigma-1Rs in the ventral striatum [18] contribute to the differential effects of self-administered cocaine on the A2AR-D2R interactions in the dorsal and ventral striatum, respectively (Figure 2). The sigma-1R is a molecular chaperone which inter alia assists in the translocation of receptors, especially D2Rs, to the plasma membrane through its binding to the receptor. It can also directly bind to the D2R in the plasma membrane [17, 91].

## 6. Conclusions

The current overview supports the hypothesis that the A2AR agonists represent novel drugs against cocaine use disorder because they inter alia can trigger the antagonistic allosteric A2AR-D2R interactions in A2AR-DR2 and A2AR-D2R-sigma-1R heteroreceptor complexes located in the ventral striatum. However, also A2AR homoreceptor complexes can participate as well as other types of A2AR heteroreceptor complexes like A2AR-mGlu5R and A2AR-mGlu5R-D2R heteroreceptor complexes [73, 92]. Such actions would result in increased activity in the ventral striatopallidal GABA antireward pathway thus leading to reward reversal and reduction of cocaine reinforcement in self-administration. The mechanism may in cocaine self-administration involve a differential reorganization of the A2AR-D2R, sigma-1R-D2R, and A2AR-D2R-sigma-1R heteroreceptor complexes and their balance with A2AR homoreceptor complexes and other types of A2AR heteroreceptor complexes in the ventral striatum as discussed above. It can lead to the differential dopaminergic signaling observed in the ventral and dorsal striatum with selective A2AR agonist induced inhibition of D2R signaling in the ventral striatum (Figure 2). The loss of the antagonistic allosteric A2AR-D2R interactions in the dorsal striatopallidal GABA neurons of the dorsal striatum found in cocaine self-stimulation can have an impact on novel habit formation versus goal-directed behavior that remains to be determined [78].
